# Impacts of Effective Microorganisms, Compost Tea, Fulvic Acid, Yeast Extract, and Foliar Spray with Seaweed Extract on Sweet Pepper Plants under Greenhouse Conditions

**DOI:** 10.3390/plants10091927

**Published:** 2021-09-15

**Authors:** Mostafa H. M. Mohamed, Rokayya Sami, Amina A. M. Al-Mushhin, Maha Mohamed Elsayed Ali, Heba S. El-Desouky, Khadiga Ahmed Ismail, Radwan Khalil, Reda M. Y. Zewail

**Affiliations:** 1 Department of Horticulture, Faculty of Agriculture, Benha University, Moshtohor, Toukh 13736, Egypt; mustafa.muhammed@fagr.bu.edu.eg; 2 Department of Food Science and Nutrition, College of Sciences, Taif University, P.O. Box 11099, Taif 21944, Saudi Arabia; 3 Department of Biology, College of Science and Humanities in Al-Kharj, Prince Sattam Bin Abdulaziz University, Al-Kharj 11942, Saudi Arabia; a.almushhin@psau.edu.sa; 4 Department of Soil and Water Sciences, Faculty of Agriculture, Benha University, Moshtohor, Toukh 13736, Egypt; maha.aly@fagr.bu.edu.eg; 5 Department of Botany, Faculty of Agriculture, Benha University, Moshtohor, Toukh 13736, Egypt; heba.alabd@fagr.bu.edu.eg (H.S.E.-D.); reda.zewail@fagr.bu.edu.eg (R.M.Y.Z.); 6 Department of Clinical Laboratory Sciences, College of Applied Medical Sciences, Taif University, P.O. Box 11099, Taif 21944, Saudi Arabia; khadigaah.aa@tu.edu.sa; 7 Botany Department, Faculty of Science, Benha University, Benha 13518, Egypt; radwan.aboelabbas@fsc.bu.edu.eg

**Keywords:** pepper, biostimulants compound, fruit yield, enzyme activity, phytohormones, fruit quality

## Abstract

Sweet pepperincludes several vitamins and is regarded as a great source of bioactive nutrients, such as carotenoids and phenolic compounds, for human growth and activities. This work aimed to investigate the effects of the soil addition of growth stimulants, namely, effective microorganisms (EM), compost tea, fulvic acid, and yeast extract, and foliar applications of seaweed extract, on the vegetative growth, enzyme activity, phytohormones content, chemical constituents of plant foliage, fruit yield, and fruit quality of sweet pepper plants *(Capsicum annuum* L. cv. Zidenka) growing under greenhouse conditions. The results showed that the tallest plant, largest leaf area/plant, and heaviest plant fresh and dry weights were recorded after combining a soil addition of yeast extract and foliar spray with seaweed extracts at 3 g/L in two growing seasons. The highest number of fruit/plant, fruit yield/m^2^, fruit values of vitamin C (VC), total sugars, total soluble solids (TSS), and carotenoids, along with the highest leaf of cytokines, P, K, Fe, and total carbohydrates values, were obtained using a soil addition of fulvic acid and spray with seaweed extract at 3 g/L in the two seasons of study. These treatments also provided the lowest abscisic acid, peroxidase, and super oxidase dismutase values in the same conditions. Sweet pepper plants supplemented with compost tea and seaweed extract foliar spray at 3 g/L were the most promising for inducing the highest values of fruit fresh and dry weights, fruit length and diameter, and the leavesrichest in N, Zn, and Mn; inversely, it induced the lowest catalase levels in both seasons. The applications of EM, yeast extract, and seaweed extract could be applied for high growth, mineral levels, enzymatic activity, fruit yield, and nutritional value of sweet pepper fruit and minimizing environmental pollution.

## 1. Introduction

Sweet pepper (*Capsicum annuum* L.) is considered to be the third most significant crop of the Solanaceae family. Pepper is one of the richest VC vegetables, and a single pepper fruit with a weight of 70 g may cover the daily VC requirements for a person. It also includes several vitamins for human growth [1]. Peppers are also regarded as a great source of bioactive nutrients, such as carotenoids and phenolics [2].

The global food security of small-scale agroecosystems accounts for almost 50% of food that is produced worldwide. These distinct agroecosystems confront many problems that mostly remain unapproachable. Meanwhile, it is considered vital to feed the anticipated growing population of humans in future years [3].

Recently, obtaining safe materials became a huge challenge for increasing human immunity and decreasing environmental pollution, especially in the current pandemic era. Biostimulant materials are described as bio-organic materials or microorganisms that are used to improve a nutrient’s absorption, stimulate growth, and stress or crop tolerance and quality [3]. Biostimulants include seaweed extracts and effective microorganisms (EM), which are active microorganisms that have lots of benefits for plant growth and development. Compost tea, fulvic acids, and yeast extract are considered principal categories and are heavily invested in. Worldwide, agricultural products based on algae are often utilized in organic or minimal input crop systems. Seaweed extract is referred to as an organic, amino acid, mineral, and vitamin nutritional source, where it canalso be considered as a vitamin precursor [3,4]. The extract from seaweed is renowned as a source of plant growth regulators because it includes large quantities of cytokinins, auxins, and gibberellins, which might encourage cell division, elongation, and differentiation [5,6]. It also enhances the development of flowers and, hence, overall fruit output. In addition, as a fresh class of agri-inputs emerging in horticulture communities and inventions, extracts that are derived from seaweed have drawn specific interest from both industrial and scientific groups [7,8].

On the other hand, EM is a commercial product that consists of a mixture of living natural cultures of microorganisms that are isolated from fertile soils and areused to increase the yield of crops and vegetables. EM also comprise aerobic and anaerobic common microorganisms, nutrients, photosynthesis, lactobacillus, Streptomyces, actinomycetes, and yeasts [9]. Efficient EM, as a biofertilizer, were used for soil application and foliar usage to stimulate growth and production. It was formed of an EM and molasses solution, which is normally added to bran or straw and then fermented to form effective microorganisms. An EM treatment was found to boost photosynthetic effectiveness and capability by increasing the availability of nutrients and the root mass [9]. Microbial diversity might involve different organic acids, antioxidants, enzymes, and chelates. EM were first chosen as an alternative to farm pesticides, but intensive investigations and field studies have established their effective uses in various areas, such as environmentally sound treatment, organic waste composting, animal manure reduction, and contaminated water treatments [10]. Using microorganisms as a soil addition improvesthe physical, chemical, and biological properties of the soil and increasesthe soil organic matter, cation exchange capacity, and available mineral nutrients; furthermore, as an environmentally friendly biofertilizer, it reduces the intensive use of expensive chemical fertilizers [11]. Compost tea has long been used in agriculture as a source of organic matter and soil amendments that provide minerals and other nutrients to plants. Compost tea is a compost extract that is made from fermented compost in water [12,13]. It consists of soluble nutrients, phytohormones, and growth regulators that are applied to the soil via irrigation systems and/or plant leaves. It also increases the phytosanitary and chemical properties of soils, which directly or indirectly influence the rhizosphere of the plant. It eliminates the pathogenic conditions of specific plants [14]. In conjunction with an integrated plant nutrition management system, the biological approaches of EM are currently gaining great traction among agriculturists and environmentalists. A growing number of rhizobial bacteria with novel properties, such as their heavy metal detoxification capacity, are being studied around the world [15]. These biofertilizers also promote plant growth by enhancing the nutrient availability to the plants, where the most studied pathways include Nfixation [16] and P and K solubilization [17]. An EMtreatment also enhances the photosynthesis rate in plants. The stomatal conductance and intracellular CO_2_ percentage were also increased [18].

Humic and fulvic acids are the most important organic matter constituents in both soils and the waste compost of municipalities. It plays an important role in the cycling of many environmental and soil ecological aspects [19]. In addition, fulvic acid is an important fraction of soil organic matter, an important portion of the dissolved organic C pool in soils, and generally shows a higher chemical and physicochemical activity compared with humic acid [20,21]. Fulvic acids also have significant functions regarding soil’s ability to buffer acids to maintain an acid–base balance. It also aids in the retention and release of metal ions and organic compounds in soil, biological availability, and mobility [16]. The purpose of suppressive composts is to increase the strength and natural suppression of land and the growth medium in agroecological systems. 

Yeast extract has a beneficial role during the vegetative growth and productivity stages through improving flower formation in some plants due to its high auxin and cytokinin contents. It also enhances carbohydrate accumulation [22]. Yeast extract also has stimulatory effects on cell division and enlargement, protein and nucleic acid synthesis, and chlorophyll formation. Furthermore, the application of yeast as a soil addition significantly increased the plant growth and yield of potato plants [23]. Therefore, the present study aimed to improve the vegetative growth, chemical constituents of plant foliage, enzyme activity, phytohormones content, fruit yield productivity, and fruit quality of sweet pepper plant cv Zidenka grown under greenhouse conditions by using a foliar spray with seaweed extract and soil addition with EM, compost tea, fulvic acid, and yeast extract.

## 2. Results and Discussion

### 2.1. Vegetative Growth Parameters

As observed in Table 1, all tested growth stimulants that were added to the soil succeeded in increasing all studied vegetative growth parameters of sweet pepper plants compared with the control plants. Regarding this issue, the soil addition of yeast extract was found to be the most effective treatment for inducing the highest values of plant height, leaf area/plant, and fresh and dry weights of leaves/plant, followed by the compost tea treatment in the two seasons. Regarding this issue, the increases in different studied vegetative growth aspects as a result of using the soil addition treatments application may have been due to the main role of such substances as a natural soil conditioner, increasing the soil-water-holding and fertility-holding capacity, chelating the nutrient elements and making them more available for absorption by plant roots, encouraging root growth, and providing a source of growth regulators, such as cytokinins, gibberellins, and auxins, causing the replacement Na with Ca and Mg on the surface of soil particles. Zaki et al. [24] showed that soil addition with EM and yeast extract at 10% improved the vegetative growth of potatoes. El-Mehy and Mohamed [25] also reported that spraying tomato plants with yeast extract significantly increased vegetative growth in terms ofthe plant height, number of branches, and fresh and dry weights of plant foliage compared with the control one. Arthur et al. [26] indicated that the soil addition of EM at 150 mL/L three times during the growing season 7 d after transplanting and at 10 d intervals was recommended to obtain a good vegetative growth of pepper plants. On the other hand, there was a positive correlation between the vegetative growth trait values and seaweed extract concentration: as the concentration of seaweed was increased, the values of plant height, leaf area, and fresh and dry weights of leaves were increased until they reached the maximum increase at the highest concentration (3 g/L). This trend was confirmed in the two seasons. In this regard, Mohammed et al. [8] and Shabana et al. [27] indicated that spraying sweet pepper with seaweed extract at 0.4% (Kelpak) during the growth of the plants significantly increased the plant height, number of branches, leaf area, and fresh and dry weights. Majeed and Marhoon [28] showed that spraying sweet pepper plants with seaweed extract at 2.5 mL/L produced a significant increase in most plant growth parameters. Moreover, Ozbay and Demirkiran [6] and Badr et al. [29] studied the effect of spraying seaweed extract at concentrations of 0, 3, or 6 mL/L on two cultivars of sweet pepper plant cv. Flavio, namely, F1 and California wonder. The results showed a significant increase in the plant height, number of branches, and the percentage of dry matter of shoots in both cultivars when treated with seaweed extract at 6 m/L as compared with the control plants.

Moreover, the data in Table 1 indicate that all interactions between growth stimulants and seaweed extract treatments increased the growth vegetative parameters of the sweet pepper plants, with significant differences as compared with the control plants in most cases in the two seasons. Specifically, the tallest plant (121.9 and 124.5 cm), largest leaf area (192.2 and 193.8 cm^2^), heaviest plant fresh weight (902 and 926 g), and plant dry weight (135 and 138.9 g) were produced by the combined treatment of the soil addition of yeast and seaweed extract at the highest concentration (3 g/L) in the first and second seasons, respectively.

### 2.2. Leaf Biochemical Composition

As tabulated in Table 2 and Table 3, it was found that the soil addition of compost tea significantly resulted in the highest values of leaf N (%), Zn (ppm), and Mn (ppm), followed by those enriched by yeast extract in the two seasons. Furthermore, the soil addition of fulvic acid statistically induced the highest values of leaf P, K, total carbohydrates (%), and Fe (ppm) in the two seasons of this study. Irrespective of the control treatment, the lowest values of leaf chemical constituent parameters were obtained by those plants that received the EM treatment in the two seasons. In this respect, the increases in macro and micronutrients concentration, mostly due to the application of plant stimulants, may have been due to their content of mineral and organic constituents, which may have affected the root growth and development, consequently increasing the absorption surface of the root to these macronutrients and, in turn, increasing its concentration in the roots and their migration, as well as the accumulation in plant foliage. In this respect, Mohammed et al. [8] and Zaki et al. [24] revealed that soil addition with EM at 10% and yeast extract at 10% enhanced the chemical constituents of potato plants. Blunden et al. [7] and El-Mehy and Mohamed [25] indicated that spraying tomato plants with yeast extract significantly increased the chemical composition of plant foliage as compared with the control plant. In addition, Badret al. [29] showed that the soil addition of EM at 150 mL/L increased leaf N, P, K, and total carbohydrate contents of tomato plants. Moreover, all applied concentrations of seaweed extract increased the leaf chemical constituent parameters in both seasons. Regarding this issue, the increase in the values of leaf chemical composition parameters was in parallel to the applied concentration of seaweed extract; hence, the seaweed extract at the high concentration produced the highest values of leaf N, P, K, total carbohydrates, Fe, Zn, and Mn in the two seasons. In this respect, Shabana et al. [27] showed that spraying sweet pepper with seaweed extract at 2.5 mL/L produced a significant increase in nutrient uptake (leaf N, P, and K content) compared with the untreated control.

Generally, the highest leaf N, Zn, and Mn values were recorded for the combined treatment with the soil addition of compost tea and foliar spraying with seaweed extract at 3 g/L, whereas the richest leaf P, K, total carbohydrates, and Fe values were recorded for those that received the soil addition of fulvic acid and sprayed with seaweed extract at 3 g/L in the two seasons.

### 2.3. Endogenous Phytohormones

**Figure 1:**
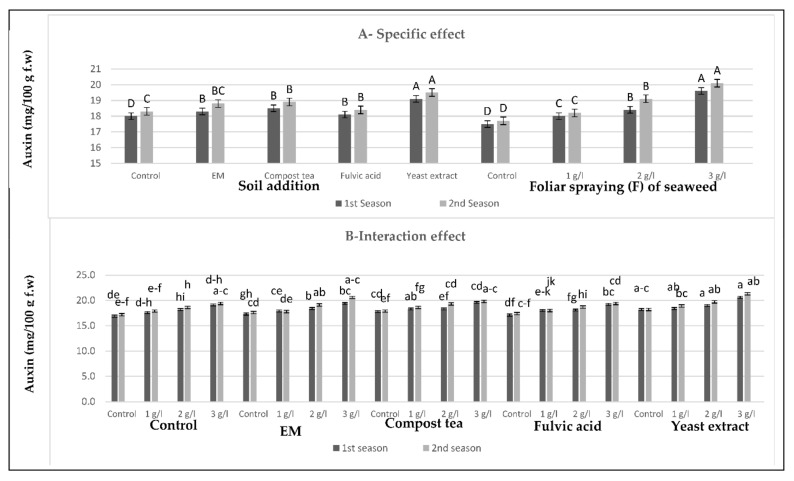
(**A**,**B**) Effects of the soil addition of EM, compost tea, fulvic acid, yeast extract, and foliar spray with seaweed extract on the auxins (mg/100 g f.w) of sweet pepper plants during the 2019/2020 and 2020/2021 seasons. Specific effects are indicated with uppercase letters and the interactions are indicated with lowercase letters, unless otherwise mentioned, according to Tukey’s multiple range test (TMRT) at *p* < 0.05.

**Figure 2:**
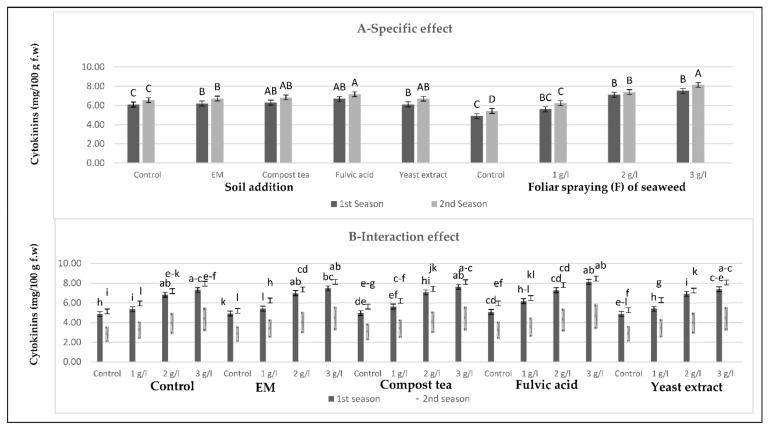
(**A**,**B**) Effects of soil addition of EM, compost tea, fulvic acid, yeast extract, and foliar spray with seaweed extract on the gibberellins (mg/100 g f.w) of sweet pepper plants during the 2019/2020 and 2020/2021 seasons. Specific effects are indicated with uppercase letters and the interactions are indicated with lowercase letters, unless otherwise mentioned, according to Tukey’s multiple range test (TMRT) at *p* ≤ 0.05.

**Figure 3:**
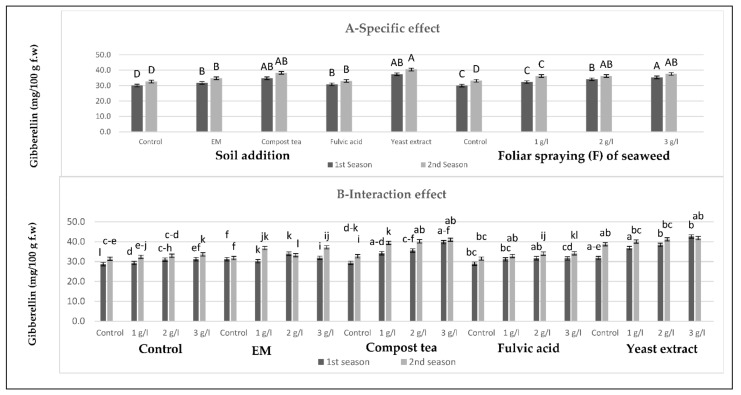
(**A**,**B**) Effects of soil addition of EM, compost tea, fulvic acid, yeast extract, and foliar spray with seaweed extract and their combinations on the cytokinins (mg/100 g f.w) of sweet pepper plants during the 2019/2020 and 2020/2021 seasons. Specific effects are indicated with uppercase letters and the interactions are indicated with lowercase letters, unless otherwise mentioned, according to Tukey’s multiple range test (TMRT) at *p* ≤ 0.05.

**Figure 4:**
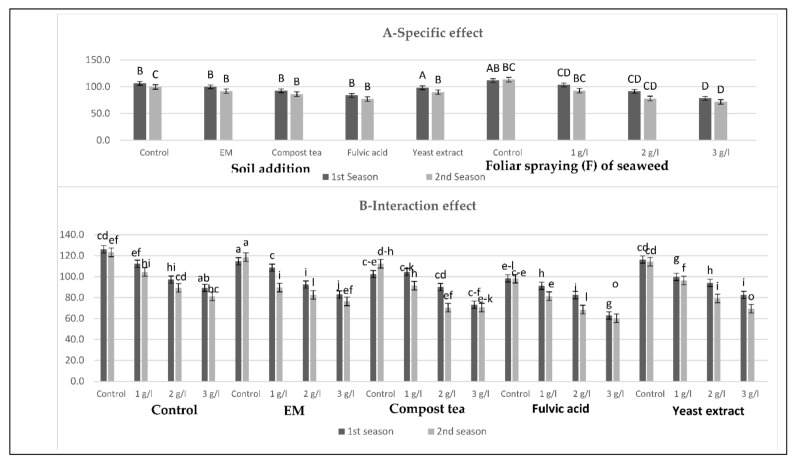
(**A**,**B**) Effects of soil addition of EM, compost tea, fulvic acid, yeast extract, and foliar spray with seaweed extract on the abscisic acid (mg/100 g f.w) of sweet pepper plants during the 2019/2020 and 2020/2021 seasons. Specific effects are indicated with uppercase letters and the interactions are indicated with lowercase letters, unless otherwise mentioned, according to Tukey’s multiple range test (TMRT) at *p* ≤ 0.05.

Figure 1, Figure 2, Figure 3 and Figure 4 show that all tested applications of growth stimulants increased the leaf auxins, gibberellins, and cytokinins contents, but it reduced the abscisic acid content as compared with the control plants in the two growing seasons. Regarding this issue, soil application of compost tea was found to be the most effective for inducing the highest leaf auxins and gibberellins contents, while the highest leaf cytokinin content, as well as the lowest leaf abscisic acid content, were registered by those who received fulvic acid treatment in the two seasons. Moreover, all spraying concentrations of seaweed extract succeeded in increasing leaf auxins, gibberellins, and cytokinins contents, but it reduced the leaf abscisic acid content in the two seasons, with greater reductions for the higher concentrations. In general, the highest leaf auxins and gibberellins contents were found for those plants who received the soil addition of yeast extract and were sprayed with seaweed extract at 3 g/L, while the highest value of leaf cytokinins content, as well as the lowest leaf abscisic acid content, were produced by the combined treatment of the soil addition of fulvic acid and seaweed extract foliar spray at 3 g/L in the two seasons.

### 2.4. Antioxidant Enzyme Activity

**Figure 5:**
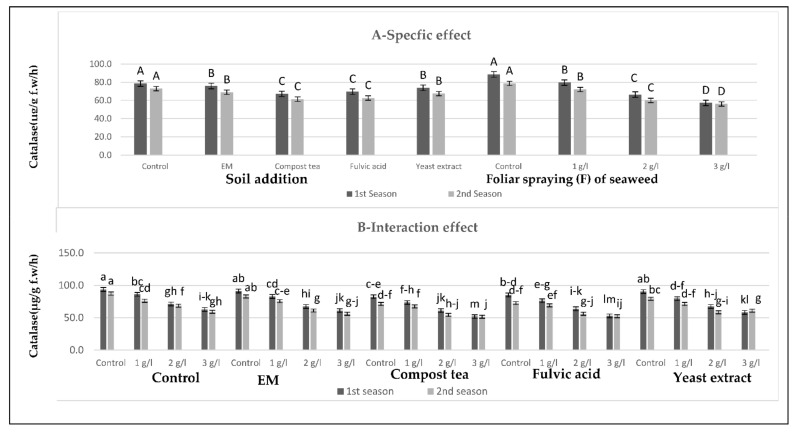
(**A**,**B**) Effects of the soil addition of EM, compost tea, fulvic acid, yeast extract, and foliar spray with seaweed extract on the catalase (µg/g f.w/h) of sweet pepper plants during the 2019/2020 and 2020/2021 seasons. Specific effects are indicated with uppercase letters and the interactions are indicated with lowercase letters, unless otherwise mentioned, according to Tukey’s multiple range test (TMRT) at *p* ≤ 0.05.

**Figure 6:**
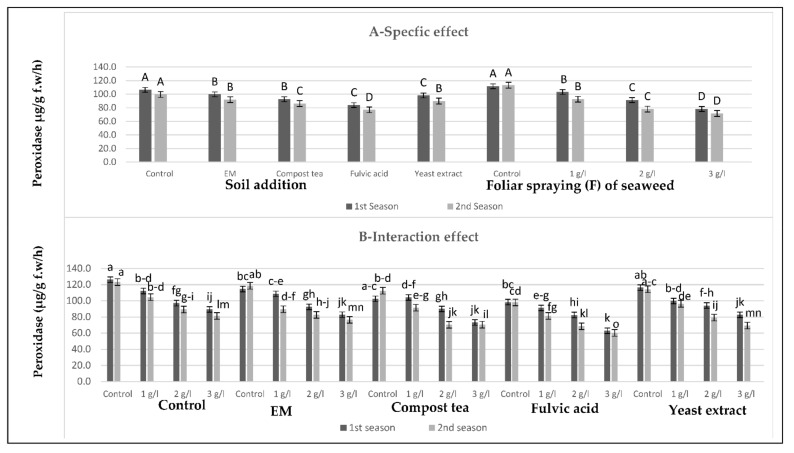
(**A**,**B**) Effects of soil addition of EM, compost tea, fulvic acid, yeast extract, and foliar spray with seaweed extract on peroxidase (µg/g f.w/h) of sweet pepper plants during the 2019/2020 and 2020/2021 seasons. Specific effects are indicated with uppercase letters and the interactions are indicated with lowercase letters, unless otherwise mentioned, according to Tukey’s multiple range test (TMRT) at *p* ≤ 0.05.

**Figure 7:**
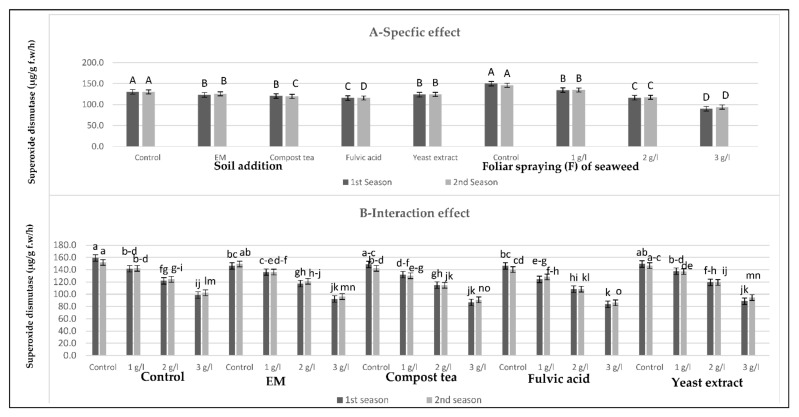
(**A**,**B**) Effects of soil addition of EM, compost tea, fulvic acid, yeast extract, and foliar spray with seaweed extract on the superoxide dismutase (µg/g f.w/h) of sweet pepper plants during the 2019/2020 and 2020/2021 seasons. Specific effects are indicated with uppercase letters and the interactions are indicated with lowercase letters, unless otherwise mentioned, according to Tukey’s multiple range test (TMRT) at *p* ≤ 0.05.

Figure 5, Figure 6 and Figure 7 show that all soil applications of growth stimulants reduced the antioxidant enzymatic activity of the sweet pepper plants in the two seasons. Regarding this issue, the soil application of fulvic acid resulted in the highest reductionsin peroxidase and superoxide dismutase, followed by the compost tea application in the two seasons. Meanwhile, the highest reduction of catalase was produced by the compost tea application, followed by fulvic acid application in the two seasons. Inversely, the highest values of the antioxidant enzymatic activity were gained by the control plants in the two seasons. On the other hand, there was a negative relationship between the antioxidant enzymatic activity trait values and the seaweed extract concentration: as the concentration of the seaweed extract increased, the values of the antioxidant enzymatic activity decreased until it reached the maximum decrease at the highest concentration of seaweed extract in the two seasons. In brief, the lowest values of peroxidase and superoxide dismutase were detected after the combined treatment of the soil addition of fulvic acid and the foliar spray with seaweed extract at 3 g/L in the two seasons. Meanwhile, the lowest value of catalase was recorded for the combined treatment of the soil addition of compost tea and seaweed extract foliar spray at the highest concentration in the two seasons. In contrast, the highest values of these parameters were produced by the control plants in the two seasons.

### 2.5. Fruit Yield

Table 4 shows that all the studied growth stimulants increased the number of fruit/plant and total fruit yield/m^2^ compared with the control plants in the two seasons. Meanwhile, the highest number of fruit/plant was produced by the fulvic acid treatment, followed by the compost tea treatment, with no significant differences between them in the two seasons. Meanwhile, the highest total fruit yield/m^2^ was achieved by the compost tea treatment, followed by the fulvic acid treatment, without significant differences between them in the two seasons. Regardless of the control treatment, the lowest number of fruit/plant and fruit yield/m^2^ were obtained by those supplemented with EM in the two seasons. Regarding this issue, such an increase in the fruit yield aspects due to soil addition with tested growth stimulants may have been due to the improvement of root growth, soil physical conditions, and increasing organic acids. This may have affected the soil pH and nutrient availability, decreasedthe number of infectionsfrom microbial disease, and increased the activity of beneficial microorganisms, which in turn positively affected the efficiency of the mineral nutrients absorption by the roots and, consequently, increased the morphological growth characteristics of the plant, which causedthe increased yield of this plant. In this regard, Zaki et al. [24] revealed that soil addition with EM and yeast extract at 10% increased the yield aspects of the potato plants. El-Mehy and Mohamed [25] reported that spraying tomato plants with yeast extract significantly increased the fruit yield as compared with the control plants. Badret al. [30] also indicated that a soil addition of EM at 150 mL/L increased the yield parameters of tomato plants. Data concerning the effect of seaweed extract concentrations on the number of fruit/plant and fruit yield/m^2^ revealed that increasing the seaweed extract concentration from 0 to 3 g/L caused a gradual increment in these parameters in both seasons. Regarding this issue, the plants sprayed with 3 g/L seaweed extract produced the highest number of fruit and fruit yield/m^2^ in the two growing seasons. Regarding this issue, Blunden et al. [7] and Arthur et al. [26] indicated that spraying sweet pepper plants with seaweed extract at 0.4% (Kelpak) significantly increased the number and size of the marketable fruit. Mohammed et al. [8] and Shabana et al. [27] also showed that spraying sweet pepper plant with seaweed extract at 2.5 mL/L produced a significant increase in fruit setting (%) and total fruit yield. Moreover, Majeed and Marhoon [28] revealed that spraying seaweed extract at concentrations of 0, 3, or 6 mL/L showed a significant increase in the number of fruit and the fruit yield in both cultivars when treated with seaweed extract at 6 mL/L. The interaction effect between the seaweed extract and growth stimulant treatments had a positive effect on the yield parameters, as the highest number of fruit/plant (16.3 and 18.6) and the highest fruit yield/m^2^ (13.92 and 15.08 kg) were produced by plants that received the fulvic acid treatment and werefoliated with the seaweed extract at 3 g/L in the two growing seasons.

### 2.6. Fruit’s Physical Quality

Table 5 shows that the sweet pepper plants that were fertilized with compost tea were found to be the most effective treatment for producing the highest values of fruit fresh and dry weights, fruit length, and diameter, followed by those enriched with the fulvic acid treatment in the two seasons. Irrespective of the control, the lowest values of these parameters were obtained by those that were augmented with EM and yeast extract in the two seasons. Furthermore, the fruit’s physical quality values of sweet pepper were greatly increased by increasing the seaweed extract concentration until the highest increase was reached at the highest concentration (3 g/L) in the two seasons. As for the interaction effect between the growth stimulants and the seaweed extract treatments, the data in Table 5 indicate that the sweet pepper plants that were supplemented with compost tea and received seaweed extract foliar spray at 3 g/L produced the highest values of fruit fresh and dry weights and length and diameter of the fruit in both seasons.

### 2.7. Fruit’s Chemical Quality

Table 6 shows that the soil addition of fulvic acid was more effective at improving the fruit’s chemical quality, i.e., the VC (mg/100g f.w), total sugars (%), TSS (%), and carotenoids (mg/100g f.w), followed by those that received compost tea, without there being a significant difference between them in the two growing seasons. Moreover, all the tested concentrations of seaweed extract increased the chemical fruit quality of sweet pepper, with the greatest increase found for the highest concentration in the two seasons. In this regard, Mohammed et al. [8] and Majeed and Marhoon [28] showed that spraying sweet pepper plants with seaweed extract at 2.5 mL/L recorded a significant increase in fruit quality as compared with control plants. In general, all tested combinations of growth stimulants and seaweed extract treatments statistically increased the fruit’s chemical quality in most cases, but it failed to induce a significant difference in the case of the total sugar parameter in both seasons. In this respect, the highest values of fruit VC (125.4 and 131.2 mg/100 g f.w), total sugars (3.71 and 3.62%), TSS (5.73 and 5.61%), and carotenoids (0.97 and 0.95 mg/100 g f.w) were achieved by those who received the combined treatment of the soil addition of fulvic acid and seaweed extract foliar spray at 3 g/L in the two seasons. It was clear from the abovementioned results that the seaweed extract increased the vegetative growth aspects, yield and its components, and chemical composition of the sweet pepper plant in comparison with the control. This was attributed to the composition of the seaweed extract, such as the natural growth hormones (auxins and cytokinins), which promote plant growth via increasing the number of metabolic events, namely, cell division and enlargement, which in turn led to increased vegetative growth aspects [31]. In addition, seaweed extract contains a considerable amount of macro- and microelements, which play an important role in the activation of many enzymes and coenzymes, which are involved in several biological processes, leading to cell division and enlargement [32,33]. These findings are in agreement with previous findings for cucumber plants [34]. It was also noted from the current results that the use of seaweed extract led to an increase in the number of branches in the treated plants, which was reflected in the increased fruit yield of the sweet pepper. This may have been due to the role of cytokinins in improving the overall growth and encouraging the growth of lateral buds and vascular tissues, and thus, increasing the number of branches and, consequently, the fruit yield. In addition, the increase in dry weight of the shoots and roots may have been associated with the increase in vegetative growth, which may have been reflected in an increase in photosynthesis and, therefore, an increase in the availability of organic nutrients, which resulted in increasing the chemical constituents [35]. Thereby, it can be recommended that foliar application with seaweed extract at 3 g/L in the presence of fulvic acid or compost tea as soil addition can be used to improve the growth, productivity, and quality of sweet pepper plants [36].

Generally, the organic fertilizers, such as compost tea, fulvic acid, and humic acid, that were used in our study generated more vegetables than those produced with inorganic fertilizers; furthermore, the organic fertilizers are more consumer-friendly since they have no synthetic substances that damage the environment or human health [37,38,39,40]. Using compost positively affects the physical and biological qualities of the soil. The heavy usage of compost may lead to plant toxicity because of the high amount of micronutrients. Applying compost is inadequate for crop requirements to be applied in the right quantity simultaneously before planting. In the growing season, adding compost tea can offer the remaining nutritional requirements for organic crops. Compost tea and EM may be produced using many techniques, either by providing aeration, utilizing the active airy extract, or using the passive airy extract. Compost tea is rich in nutrients, organic compounds, and helpful bacteria, which also enhance the physical and chemical qualities of the soil and inhibit some diseases [40,41,42,43].

The novelty of using the biostimulants in agriculture has been researched for a long time but has only recently received interest as a strategy to ease the detrimental impact of climate change on agriculture and to increase plant growth and defense systems in the face of diverse stresses [40]. The successful use of biostimulants involves utilizing them in the form of phytochemical combinations that might increase the growth and yield while improving biotic and abiotic stress protection without disadvantages.

## 3. Materials and Methods

Two field experiments were performed during the two successive growing seasons of 2019/2020 and 2020/2021 in a private farm in El-Khatatba village, Monufia Governorate, Egypt (30°31′05″N and 30°07′34″E) to investigate the effects of the soil addition of some growth stimulants, namely, EM, compost tea, fulvic acid, and yeast extract, as well as foliar spraying with seaweed extract, on the vegetative growth and chemical composition of plant foliage. In addition, fruit yield and the quality of sweet pepper plants (*Capsicum annum* L. cv. Zidenka) grown under greenhouse conditions were measured. Soil samples were randomly taken from the top 30 cm of the soil surface and physical and chemical analyses were undertaken [30,44]. Briefly, the texture was determined using a pipette. Soil electrical conductivity and pH were examined in a suspension of 1:2.5 (soil:water*w*:*v*), while the cations and anions were estimated in a soil paste. Available values of N, P, and K were determined after their extraction using KCl (1M), NaHCO_3_ (0.5 M, pH 8.5), and CH_3_COONH_4_ (1M, pH 7). The soil’s physical and chemical properties are presented in Table 7.

### 3.1. Plant Materials and Experimental Layout

The Zidenka red sweet pepper was the variety that was used for this investigation. The Zidenka’s seeds were sourced from the Holland Corporation Rijk Zwaan. The seeds were sowed on 1August in seedling trays with 209 holes during the two experimental seasons. Forty-five days after planting the seeds, the seedlings with 2–3 real leaves were transported to the plastic greenhouse (40 m length × 9 m width × 3.5 m height) and cultivated on both sides of the row, where the distance between transplants was 40 cm. The area of the experimental plot was 10 m^2^; it contained 1 line that was 10 m in length and 1 m in width. The Spanish pruning method was used.

The experimental design was a factorial experiment in a complete randomized block design (CRBD) with 20 treatments represented with the combination of two factors; the first factor was the soil addition of a growth stimulant, whereas the second factor was the concentration of a foliar spray with seaweed extract (5 soil additions × 4 foliar sprays), replicated three times.

### 3.2. Treatments

#### 3.2.1. Soil Addition of Some Growth Stimulant Treatments

The first treatmentwas the control (without any soil addition of growth stimulants), the second was EM at 5 L/100 m^2^, the third was a compost tea at 10 L/100 m^2^, the fourth was fulvic acid at 5 kg/100 m^2^, and the fifth was yeast extract at 10 L/100 m^2^.

Contents and characteristics of the soil addition treatments

EM is a commercial name that consists of a mixture of living natural cultures of microorganisms isolated from fertile soils and is used to increase the yield of crops and vegetables. It was obtained from the Egyptian Ministry of Agriculture and Land Reclamation. It included photosynthesis bacteria (*Rhodopseudomonaspalustrus* and *Rhodobacter space*), milk bacteria (*Lactobacillus casei* and *Streptococcus lactis*), yeasts (*Saccharomyces albus* and *Candida utilis*), actinomycetes (*Streptomyces albus* and *Streptomyces griseus*) and *molds* (*Aspergillusoryzae* and *Mucomhiemalis*) [45]. EM also comprise common aerobic and anaerobic microorganisms, nutrients, photosynthesis, lactobacillus bacteria, streptomyces, actinomycetes, and yeasts [18].

Compost tea preparation: It was prepared by soaking 10 kg of mature plant compost with 100 L of water + 100 mL molasses for 7 days in a special unit, which was attached to an air pump, where the aerator provided a continuous flow of air bubbles to extract the compost tea until the completion of the fermentation process and extract color became light brown [46].

Chemical and microbiological analyses of the compost tea produced the following values: pH 7.02, EC 2.12dS/m, total N 0.38%, total P 0.09%, total K 0.41%, total count of bacteria 8.1 × 10^6^cfu/mL, total count of fungi 7.8 × 10^4^cfu/mL, and total count of actinomycetes 1.6 × 10^5^cfu/mL.

Yeast extract preparation: A technique that allowed for yeast cells (commercial soft yeast) to be efficiently grown and multiplied during conductive aerobic and nutritional conditions was used. It also produced denovo beneficial bio-constituents (i.e., carbohydrates, sugars, proteins, amino acids, fatty acids, hormones, etc.). Thus, it allowed for such constituents to be releasedfrom the yeast cells in a readily usable form using two consecutive cycles of thawing and freezing. Such a technique for yeast preparation based on nutritional medium glucose and casein has 2 high-quality sources of C, N, and other essential elements in a suitable balance. Air pumping and adjusting the incubation freezing temperature were used for the disruption of yeast cells and releasing their content.

Chemical analysis of the prepared yeast extract stock solution was produced the following results: total protein (5.3%), total carbohydrates (4.7%), N (1.2%), P (0.13%), K (0.3%), Mg (0.013%), Ca (0.02%), and Na (0.01%); the micro-elements were Fe (0.13 ppm), Mn (0.07 ppm), Zn (0.04 ppm), Cu (0.04 ppm), 3 (0.016 ppm), Mo (0.0003 ppm), IAA (0.5 μg/mL), and GA (0.3 μg/mL) [47,48].

#### 3.2.2. Foliar Spraying Treatments

Foliar spraying treatments: control (spraying with tap water only), seaweed extract at 1 g/L, seaweed extract at 2 g/L, and seaweed extract at 3 g/L.

Seaweed extract composition and characteristics: commercial seaweed extract product “Alga 600” (Technogreen), which is a mixture of three seaweeds, viz., *Ascophyllumnodosum*, *Laminariaspp*, and *Sargassum sp*. The seaweed extract also contained N (1%), K (18.5%), Ca (0.17%), Mg (0.42%), Fe (0.06%), S (2.2%), alginic acids (10–12%), and plant hormones (600 ppm) [6].

Soil addition treatments were applied five times, starting 15 days from transplanting and every two weeks thereafter, while the spray treatments were applied 10 times, starting 30 days from transplanting and every two weeks thereafter through the growing season. All other agricultural practices concerning cultivation, fertilization, irrigation, pest, diseases, and weed control were conducted as commonly followed according to the recommendation of the Egyptian Ministry of Agriculture for the commercial production of sweet pepper plants under greenhouse conditions.

### 3.3. Data Collection and Recording

Data of the vegetative growth, chemical constituents of plant foliage, yield and its components, andthe fruit’s physical and chemical qualities were recorded as follows.

#### 3.3.1. Vegetative Growth Characteristics

Three plants were randomly taken from each experimental plot as a representative sample 75 days after transplanting and the following data were recorded:plant length, average leaf area, and fresh and dry weights per plant.

#### 3.3.2. Chemical Analysis of Plant Foliage

The total nitrogen (%) was determined in the digested dry matter of plant leaves was determined using a microKyldahel [49]; the phosphorus content was determined using a spectrophotometer method [50]; the potassium content was determined using the flame photometer method [51]; the total carbohydrates were determined from the digested dry matter of plant leaves [52]; and the Fe, Zn, and Mn levels were determined using an atomic absorption spectrophotometer [53].

#### 3.3.3. Estimation of Endogenous Phytohormones

Endogenous phytohormones in sweet pepper leaves at the blooming stage were quantified using high-performance liquid chromatography in both seasons (HPLC). For the hormonal analysis, 10 g of fresh weight leaves were chopped into small pieces and macerated before being extracted twice with 96 percent methanol, then again with 40 percent methanol for 24 h [54]. The methanolic extract was filtered and evaporated into an aqueous solution in a rotary evaporator at 40 °C. The solution was adjusted to a pH of 8.6 before being extracted four times with 100 mL of ethyl acetate. The alkaline ethyl acetate solution was combined and purified with one teaspoon of anhydrous sodium sulphate per 100 mLThe ethyl acetate fraction was filtered and evaporated to dryness, and the residue was dissolved in 4 mL pure methanol. According to [55], this extraction was utilized to determine cytokinin levels. The aqueous solution was acidified to pH 2.6–2.8 and extracted as stated above; this extraction was utilized to determine gibberellic acid (GA3), indole-3-acetic acid (IAA), and abscisic acid (ABA) using an HPLC according to the procedures mentioned above by [54]. The phytohormones were identified by comparing the peak retention times to the retention periods of genuine compounds. The quality of specific plant hormones was evaluated by comparing the peak area generated by a known weight of plant material to the standard curves of legitimate chemicals, which represented the connection between different concentrations and their peak areas. All endogenous phytohormone findings were expressed as milligrams/100 g fresh weight for auxins (IAA), gibberellins, and abscisic acid (ABA) [54,56].

#### 3.3.4. Assay of Enzyme Activity

Preparation of enzyme extract

Samples of plant leaves were ground with a 0.2 M Tris HCl buffer (pH 7.8) containing 14 mM β-mercaptoethanol in the ratio of 1/3 *w*/*v*. The extracts were centrifuged at 10,000 rpm for 20 min at 4 °C. The supernatant was used to determine the activities of the following oxidative enzymes [57].

Catalase (CAT) activity

Catalase was measured spectrophotochemically as described in [58]. The enzyme extract (100 L) was mixed with 100 L of 100 mM H2O2, and the total volume was increased to 1 mL using a 250 mM phosphate buffer at pH 6.8. Every minute, the optical density at 240 nm was measured in comparison to a blank.

Peroxidase activity (POX) activity

The peroxidase test (based on the oxidation of pyrogallol to purpurogallin in the presence of H2O2) was performed as reported by [57]. 0.5 mL of 0.1 M potassium phosphate buffer solution at pH 7.0, 0.3 mL enzyme extract, 0.3 mL 0.05 M pyrogallol, and 0.1 mL 1.0 percent H2O2 were added to the reaction mixture. The mixture was finished with 3 mL of distilled water. In a control blank cuvette, the enzyme extract was replaced with distilled water. The absorbance of 1 mL was measured, and the peroxidase activity was calculated as the difference in absorbance at 425 nm/15 min/gram fresh weight.

Superoxide dismutase (SOD) activity

In an ice bucket, fresh leaf samples were gathered and transported to the laboratory. After that, the leaves were rinsed with distilled water and the surface moisture was brushed away. With a pre-chilled pestle and mortar, the leaf samples (0.5 g) were homogenized in an ice-cold 0.1 M phosphate buffer (pH 7.5) containing 0.5 mM EDTA. The homogenate was transferred to centrifuge tubes and spun for 15 min at 15,000 g in a Beckman refrigerated centrifuge at 4 °C. The enzyme extract was created by transferring the supernatant to 30 mL tubes. SOD activity was calculated by measuring the reduction in absorbance of the superoxide–nitro blue tetrazolium complex using the enzyme. [57]. From each enzyme sample, 3 mL of reaction mixture containing 0.1 mL of 1.5 M sodium carbonate, 0.2 mL of 200 mM methionine, 0.1 mL of 2.25 mM nitroblue tetrazolium, 0.1 mL of 3 mM EDTA, 1.5 mL of 100 mM potassium phosphate buffer, 1 mL pure water, and 0.05 mL of the enzyme was taken in test as controls, two tubes were obtained that did not contain the enzyme extract. The reaction was initiated by adding 0.1 mL riboflavin (60 M) and putting the tubes beneath two 15 W fluorescent lights for 15 min. By turning off the light and covering the tubes with a dark cloth, the reaction was halted. The most colour was produced in the tubes that did not contain the enzyme. As a control, a non-irradiated full reaction mixture that did not develop colour was used. The absorbance was measured at 560 nm, and one unit of enzyme activity was defined as the amount of enzyme that lowered the absorbance reading of samples by 50% when compared to tubes without enzymes [58].

#### 3.3.5. Fruit Yield Parameters and Their Components

Fruit yield (kg/m^2^) was calculated from the fruit yield/plant and the number of plants/m^2^; the number of fruit/plant was also calculated.

#### 3.3.6. Physical Fruit Quality

A random sample of 10 fruit at a suitable maturity stage from each experimental plot was taken to determine the fresh and dry weights of the fruit, fruit length, and diameter.

#### 3.3.7. Chemical Fruit Quality Estimations

Total soluble solids (TSS) were determined using a hand refractometer.

Carotenoids:Total carotenoids were determined by soaking 1 g of the fresh fruit in 5 mL of methanol for 2 h at room temperature under dark conditionsin order toobtain complete extraction. The carotene was separated using hexane through a separating funnel. The volume was made up to 10 mL with hexane and then this layer was again passed through sodium sulfonate through a funnel in order to remove any moisture from the layer. The absorbance of the layer was measured spectrophotometrically at 436 nm (using hexane as a blank) [59].

Ascorbic acid (VC) was determined by using the indicator of 2,6-dichlorophenolindophenol via titration, where the total sugars in fresh samples of ripe fruit for each experimental plot were calorimetrically determined [59,60].

### 3.4. Statistical Analysis

The collected data were analyzed using the ANOVA (CROPSTAT 2007.2) technique and determined using Tukey’s multiple range test (*p* < 0.05).

## 4. Conclusions

In general, adding EM, yeast extract, fulvic acid, and compost tea as soil additions separately or incombination with seaweed as a foliar application at the two levels enhanced the sweet pepper development features and biochemical components (mineral elements and some bio-constituents, such as endogenous phytohormones and enzyme activity). Furthermore, the use of these treatments improved the fruit quality of sweet pepper, including the fruit fresh and dry weight, fruit diameter, and fruit length. Furthermore, the nutritional quality of the sweet pepper was improved, including the TSS, VC, total sugars, and carotenoids. The new aspect of this study was the improvement in production and fruit quality, as well as the reduction in environmental pollutants, which was achieved by the application of biostimulant treatments that are both safe and ecologically benign.

## Figures and Tables

**Table 1:** Effects of the soil addition of EM, compost tea, fulvic acid, yeast extract, and foliar spray with seaweed extract and their combinations on the vegetative growth of sweet pepper plants during the 2019/2020 and 2020/2021 seasons.

Treatments		Plant Height (cm)		Average Leaf Area/Plant (cm^2^)		Plant Fresh Weight (g)		Plant Dry Weight (g)	
Soil Addition	Foliar Spraying	1st Season	2nd Season	1st Season	2nd Season	1st Season	2nd Season	1st Season	2nd Season
Control		101.3 ^B^	105.5 ^C^	173.8 ^B^	166.4 ^D^	798 ^D^	772 ^D^	112.9 ^B^	104.6 ^B^
EM		115.1 ^A^	114.8 ^AB^	175.4 ^B^	173.9 ^C^	806 ^C^	807 ^C^	113.0 ^B^	109.4 ^B^
Compost tea		117.9 ^A^	116.1 ^AB^	184.8 ^A^	178.9 ^B^	849 ^B^	830 ^B^	118.9 ^AB^	112.5 ^A^
Fulvic acid		105.8 ^B^	110.5 ^BC^	176.6 ^B^	176.3 ^BC^	810 ^C^	818 ^BC^	113.4 ^AB^	110.8 ^A^
Yeast extract		119.4 ^A^	120.1 ^A^	186.4 ^A^	187.1 ^A^	857 ^A^	861 ^A^	120.0 ^A^	117.8 ^B^
	Control	108.9 ^B^	109.4 ^B^	174.5 ^B^	171.6 ^B^	783 ^D^	771 ^D^	101.9 ^D^	92.6 ^D^
	Seaweed at 1g/L	110.9 ^AB^	111.3 ^B^	175.2 ^B^	173.8 ^B^	804 ^C^	797 ^C^	111.4 ^C^	103.6 ^C^
	Seaweed at 2g/L	113.1 ^AB^	116.0 ^A^	182.0 ^A^	179.8 ^A^	836 ^B^	842 ^B^	118.5 ^B^	117.8 ^B^
	Seaweed at 3g/L	114.8 ^A^	116.8 ^A^	186.2 ^A^	181.0 ^A^	873 ^A^	866 ^A^	130.7 ^A^	129.9 ^A^
Control	Control	98.6 ^d^	102.3 ^e^	168.2 ^e^	162.4 ^i^	756 ^k^	729 ^k^	98.3 ^i^	87.5 ^j^
	Seaweed at 1g/L	99.3 ^cd^	104.2 ^de^	169.9 ^de^	164.2 ^hi^	777 ^ij^	754 ^jk^	104.9 ^e–i^	98.1 ^g^
	Seaweed at 2g/L	104.3 ^a–d^	108.3 ^b–e^	178.1 ^a–e^	169.9 ^f–i^	818 ^fg^	794 ^fi^	122.6 ^b–f^	111.2 ^fg^
	Seaweed at 3g/L	103.2 ^b–d^	107.1 ^c–e^	179.6 ^a–e^	169.3 ^g–i^	841 ^de^	811 ^d–g^	126.1 ^b–f^	121.6 ^c–e^
EM	Control	112.1 ^a–d^	111.6 ^a–e^	171.0 ^d–e^	169.3 ^g–i^	769 ^jk^	761 ^i–k^	99.9 ^hi^	91.3 ^ij^
	Seaweed at 1g/L	114.2 ^a–d^	113.2 ^a–e^	169.8 ^d–e^	171.4 ^e–h^	781 ^ij^	786 ^g–j^	109.0 ^d–h^	102.3 ^gh^
	Seaweed at 2g/L	116.3 ^a–c^	116.1 ^a–e^	174.9 ^b–e^	176.8 ^c–g^	801 ^gh^	827 ^c–f^	112.2 ^c–g^	115.8 ^de^
	Seaweed at 3g/L	118.0 ^ab^	118.3 ^a–d^	186.2 ^a–c^	178.2 ^c–f^	874 ^b^	854 ^b–c^	131.0 ^a–e^	128.2 ^bd^
	Control	114.5 ^ad^	113.1 ^a–e^	178.6 ^a–e^	176.0 ^e–g^	801 ^gh^	792 ^f–i^	104.3 ^f–i^	95.1 ^hi^
Compost tea	Seaweed at 1g/L	116.8 ^a–c^	112.4 ^a–e^	181.0 ^a–e^	175.8 ^c–g^	832 ^ef^	805 ^e–h^	116.1^c–g^	104.6 ^ef^
	Seaweed at 2g/L	119.3 ^ab^	118.6 ^a–d^	189.2 ^ab^	181.6 ^c–g^	871 ^bc^	851 ^bc^	121.6^a–d^	119.2 ^bc^
	Seaweed at 3g/L	121.3 ^a^	120.1 ^a–c^	190.6 ^a^	182.2 ^c^	893 ^a^	873 ^b^	133.6 ^a^	131.0 ^a^
Fulvic acid	Control	103.2 ^b–d^	104.6 ^de^	173.2 ^c–e^	171.1 ^e–h^	778 ^ij^	769 ^h–i^	101.2 ^g–i^	92.4 ^ij^
	Seaweed at 1g/L	106.2 ^a–d^	109.3 ^b–e^	172.3 ^c–e^	173.4 ^d–g^	791 ^hi^	795 ^f–i^	110.0 ^e–i^	103.4 ^ab^
	Seaweed at 2g/L	104.3 ^a–d^	114.2 ^a–e^	178.6 ^a–e^	179.3 ^c–e^	819 ^fg^	841 ^b–e^	114.2 ^a–e^	117.4 ^de^
	Seaweed at 3g/L	109.8 ^a–d^	114.1 ^a–e^	182.3 ^a–e^	181.6 ^cd^	855 ^bc^	868 ^b^	128.2 ^ab^	130.2 ^ab^
Yeast extract	Control	116.2 ^a–d^	115.6 ^a–e^	181.3 ^a–e^	179.3 ^b–e^	814 ^fg^	805 ^e–h^	105.9 ^g–i^	96.6 ^ij^
	Seaweed at 1 g/L	118.3 ^ab^	117.3 ^a^	183.2 ^a–d^	184.3 ^bc^	841 ^de^	846 ^b–d^	117.2 ^d–i^	110.0 ^gh^
	Seaweed at 2 g/L	121.2 ^a^	123.0 ^ab^	189.2 ^ab^	191.3 ^ab^	874 ^b^	897 ^b^	122.1 ^a–e^	125.6 ^de^
	Seaweed at 3 g/L	121.9 ^a^	124.5 ^a^	192.2 ^a^	193.8 ^a^	902 ^a^	926 ^a^	135.0 ^a–c^	138.9 ^de^

Means between treatments in the same column followed by the same letter were not significantly different according to Tukey’s multiple range test (TMRT) at *p* ≤ 0.05. Specific effects are identified with uppercase letters and the interactions are identified with lowercase letters unless otherwise mentioned.

**Table 2:** Effects of soil addition of EM, compost tea, fulvic acid, yeast extract, and foliar spray with seaweed extract and their combinations on the chemicalconstituents of the plant foliage of sweet pepper plants during the 2019/2020 and 2020/2021 seasons.

Treatments		Nitrogen (%)		Phosphorus (%)		Potassium (%)		Total Carbohydrates (%)	
Soil Addition	Foliar Spraying	1st Season	2nd Season	1st Season	2nd Season	1st Season	2nd Season	1st Season	2nd Season
Control		2.36 ^C^	2.32 ^D^	0.229 ^E^	0.233 ^E^	1.45 ^D^	1.47 ^C^	17.9 ^D^	18.8 ^D^
EM		2.39 ^C^	2.37 ^D^	0.237 ^D^	0.238 ^D^	1.51 ^C^	1.69 ^B^	19.1 ^C^	21.3 c
Compost tea		2.52 ^A^	2.59 ^A^	0.240 ^C^	0.242 ^C^	1.74 ^A^	1.81 ^A^	22.6 ^A^	24.4 ^A^
Fulvic acid		2.45 ^B^	2.46 ^C^	0.263 ^A^	0.269 ^A^	1.78 ^A^	1.86 ^A^	23.4 ^A^	25.0 ^A^
Yeast extract		2.47 ^AB^	2.53 ^B^	0.259 ^B^	0.263 ^B^	1.67 ^B^	1.73 ^B^	20.3 ^B^	23.4 ^B^
	Control	2.33 ^C^	2.34 ^D^	0.230 ^D^	0.235 ^D^	1.53 ^D^	1.59 ^C^	18.7 ^C^	20.2 ^C^
	Seaweed at 1 g/L	2.41 ^B^	2.43 ^C^	0.241 ^C^	0.242 ^C^	1.58 ^C^	1.69 ^B^	20.1 ^B^	22.2 ^B^
	Seaweed at 2 g/L	2.49 ^A^	2.49 ^B^	0.251 ^B^	0.255 ^B^	1.68 ^B^	1.75 ^A^	21.6 ^A^	23.7 ^A^
	Seaweed at 3 g/L	2.51 ^A^	2.56 ^A^	0.258 ^A^	0.262 ^A^	1.72 ^A^	1.80 ^A^	22.3 ^A^	24.1 ^A^
Control	Control	2.29 ^f^	2.25 ^j^	0.217 ^l^	0.223 ^i^	1.34 ^i^	1.39 ^h^	16.7 ^i^	17.2 ^j^
	Seaweed at 1 g/L	2.36 ^e–f^	2.29 ^hi^	0.226 ^k^	0.229 ^hi^	1.39 ^i^	1.42 ^gh^	17.2 ^hi^	19.3 ^h–j^
	Seaweed at 2 g/L	2.39 ^e–f^	2.36 ^f–i^	0.234 ^ij^	0.239 ^ef^	1.54 ^gh^	1.51 ^f–h^	18.9 ^f–i^	19.1 ^ij^
	Seaweed at 3 g/L	2.38 ^e–f^	2.41 ^e–h^	0.237 ^h–j^	0.238 ^e–g^	1.53 ^gh^	1.53 ^f–h^	18.7 ^f–i^	19.6 ^g–i^
EM	Control	2.31 ^f^	2.29 ^hi^	0.225 ^k^	0.230 ^g–i^	1.41 ^i^	1.54 ^fg^	17.3 ^hi^	19.3 ^h–j^
	Seaweed at 1 g/L	2.41 ^d–f^	2.37 ^f–i^	0.239 ^f–i^	0.241 ^de^	1.52 ^h^	1.65 ^d–f^	19.8 ^d–i^	21.8 ^d–g^
	Seaweed at 2 g/L	2.39 ^ef^	2.42 ^d–h^	0.238 ^g–j^	0.238 ^e–g^	1.51 ^h^	1.78 ^b–d^	19.2 ^e–i^	21.6 ^e–h^
	Seaweed at 3 g/L	2.42 ^c–f^	2.40 ^e–h^	0.245 ^d–g^	0.241 ^de^	1.59 ^f–h^	1.76 ^cd^	20.0 ^d–g^	22.4 ^d–f^
Compost tea	Control	2.38 ^ef^	2.42 ^d–h^	0.231 ^jk^	0.234 ^e–h^	1.62 ^e–g^	1.69 ^c–e^	19.8 ^d–h^	21.6 ^e–h^
	Seaweed at 1 g/L	246 ^b–f^	2.53 ^c–e^	0.242 ^e–h^	0.232 ^f–h^	1.69 ^c–e^	1.78 ^b–d^	21.8 ^b–e^	23.8 ^c–e^
	Seaweed at 2 g/L	2.59 ^a–c^	2.65 ^a–c^	0.240 ^f–i^	0.249 ^cd^	1.80 ^ab^	1.83 ^a–c^	23.8 ^a–c^	25.8 ^a–c^
	Seaweed at 3 g/L	2.64 ^a^	2.75 ^a^	0.246 ^d–h^	0.251 ^c^	1.84 ^a^	1.92 ^ab^	24.9 ^a^	26.2 ^ab^
Fulvic acid	Control	2.33 ^f^	2.34 ^g–i^	0.241 ^e–i^	0.248 ^cd^	1.68 ^d–f^	1.73 ^c–e^	21.3 ^c–f^	22.4 ^d^
	Seaweed at 1 g/L	2.39 ^ef^	2.49 ^d–f^	0.252 ^d^	0.256 ^c^	1.72 ^b–d^	1.82 ^bc^	21.9 ^b–d^	24.1 ^c^
	Seaweed at 2 g/L	2.57 ^a–d^	2.46 ^d–g^	0.273 ^bc^	0.279 ^b^	1.83 ^a^	1.91 ^ab^	24.3 ^ab^	26.3 ^ab^
	Seaweed at 3 g/L	2.51 ^a–e^	2.53 ^c–e^	0.286 ^a^	0.292 ^a^	1.86 ^a^	1.97 ^a^	26.1 ^a^	26.9 ^a^
Yeast extract	Control	2.32 ^f^	2.38 ^f–i^	0.238 ^g–j^	0.242 ^de^	1.59 ^f–h^	1.61 ^ef^	18.2 ^g–i^	20.3 ^f–i^
	Seaweed at 1 g/L	2.41 ^d-f^	2.48 ^d–g^	0.248 ^de^	0.251 ^c^	1.58 ^gh^	1.76 ^cd^	19.6 ^d–h^	21.9 ^d–g^
	Seaweed at 2 g/L	2.52 ^a–e^	2.56 ^bc^	0.268 ^c^	0.272 ^b^	1.73 ^b–d^	1.74 ^c–e^	21.7 ^b–e^	25.8 ^a–c^
	Seaweed at 3 g/L	2.60 ^ab^	2.70 ^ab^	0.279 ^ab^	0.286 ^a^	1.78 ^a–c^	1.80 ^bc^	21.6 ^c–e^	25.4 ^a–c^

Means between treatments in the same column followed by the same letter were not significantly different according to Tukey’s multiple range test (TMRT) at *p* ≤ 0.05. Specific effects are indicated with uppercase letters and the interactions are indicated with lowercase letters unless otherwise mentioned.

**Table 3:** Effects of the soil addition of EM, compost tea, fulvic acid, yeast extract, and foliar spray with seaweed extract and their combinations on the micronutrients content of the plant foliage of sweet pepper plants during the 2019/2020 and 2020/2021seasons.

Treatments		Fe (ppm)		Zn (ppm)		Mn (ppm)	
Soil Addition	Foliar Spraying	1st Season	2nd Season	1st Season	2nd Season	1st Season	2nd Season
Control		110.2 ^C^	117.3 ^C^	30.6 ^C^	31.3 ^C^	43.2 ^C^	41.7 ^D^
EM		112.2 ^BC^	123.3 ^B^	30.7 ^B^	33.5 ^AB^	44.8 ^B^	42.9 ^CD^
Compost tea		113.8 ^B^	124.3 ^B^	32.3 ^B^	32.8 ^BC^	46.7 ^A^	45.6 ^A^
Fulvic acid		116.4 ^A^	128.7 ^A^	31.3 ^BC^	32.4 ^BC^	45.6 ^AB^	44.7 ^AB^
Yeast extract		112.7 ^B^	122.0 ^B^	34.4 ^A^	35.1 ^A^	45.3 ^B^	43.5 ^BC^
	Control	94.0 ^D^	106.7 ^D^	26.7 ^D^	27.6 ^D^	38.0 ^D^	36.1 ^D^
	Seaweed at 1 g/L	111.9 ^C^	118.5 ^C^	30.5 ^C^	31.1 ^C^	43.2 ^C^	41.9 ^C^
	Seaweed at 2 g/L	119.8 ^B^	127.5 ^B^	34.6 ^B^	34.8 ^B^	48.0 ^B^	46.6 ^B^
	Seaweed at 3 g/L	126.7 ^A^	139.6 ^A^	37.1 ^A^	38.5 ^A^	51.2 ^A^	50.2 ^A^
Control	Control	92.3 ^k^	96.8 ^n^	24.7 ^k^	26.2 ^j^	36.8 ^j^	34.1 ^i^
	Seaweed at 1 g/L	108.2 ^j^	116.2 ^i–k^	28.3 ^g–k^	29.4 ^f–j^	39.2 ^ij^	38.2 ^gh^
	Seaweed at 2 g/L	116.3 ^f–h^	123.2 ^f–h^	34.0 ^c–e^	33.5 ^c–g^	47.2 ^f–g^	46.2 ^c–e^
	Seaweed at 3 g/L	124.1 ^a–d^	132.9 ^c–e^	35.7 ^a–c^	36.2 ^b–d^	49.5 ^a–e^	48.4 ^a–d^
EM	Control	92.9 ^k^	108.6 ^lm^	27.2 ^h–k^	28.6 ^h–j^	38.0 ^j^	35.8 ^hi^
	Seaweed at 1 g/L	110.4 ^ij^	119.6 ^h–j^	31.6 ^d–g^	31.8 ^d–i^	41.8 ^hi^	39.8 ^gh^
	Seaweed at 2 g/L	119.6 ^d–f^	128.7 ^e–f^	34.8 ^b–d^	34.6 ^b–e^	48.4 ^b–f^	46.1 ^de^
	Seaweed at 3 g/L	126.0 ^a–c^	136.2 ^b–d^	36.2 ^a–c^	38.9 ^ab^	51.0 ^a–c^	49.8 ^a–d^
Compost tea	Control	93.8 ^k^	112.2 ^kl^	26.4 ^i–k^	27.2 ^i–j^	39.4 ^ij^	37.4 ^g–i^
	Seaweed at 1 g/L	113.2 ^g–j^	117.3 ^h–k^	30.4 ^e–h^	29.8 ^e–j^	46.3 ^e–g^	45.8 ^de^
	Seaweed at 2 g/L	121.2 ^c–f^	126.4 ^e–g^	34.9 ^b–d^	36.0 ^b–d^	48.1 ^c–f^	47.6 ^a–e^
	Seaweed at 3 g/L	127.2 ^ab^	141.3 ^b^	37.6 ^ab^	36.5 ^bd^	52.8 ^a^	51.6 ^a^
Fulvic acid	Control	96.7 ^k^	114.3 ^j–l^	25.6 ^jk^	26.8 ^j^	38.2 ^j^	37.1 ^hi^
	Seaweed at 1 g/L	115.6 ^f–i^	121.8 ^g–i^	28.9 ^g–j^	31.8 ^d–i^	45.0 ^f–h^	44.2 ^ef^
	Seaweed at 2 g/L	123.8 ^b–e^	130.4 ^de^	34.2 ^b–e^	34.2 ^b–f^	47.3 ^d–f^	46.8 ^b–e^
	Seaweed at 3 g/L	129.8 ^a^	148.6 ^a^	36.8 ^a–c^	36.9 ^bc^	51.9 ^ab^	50.8 ^ab^
Yeast extract	Control	94.3 ^k^	102.0 ^mn^	29.8 ^f–i^	29.3 ^g–j^	37.4 ^j^	36.2 ^hi^
	Seaweed at 1 g/L	112.4 ^h–j^	118.0 ^h–k^	33.2 ^c–f^	32.8 ^c–h^	43.7 ^gh^	41.3 ^fg^
	Seaweed at 2 g/L	118.2 ^e–g^	128.6 ^ef^	35.1 ^bc^	35.7 ^b–d^	49.2 ^b–e^	46.4 ^c–e^
	Seaweed at 3 g/L	126.4 ^a–c^	139.4 ^bc^	39.4 ^a^	42.7 ^a^	50.8 ^a–d^	50.2 ^a–c^

Means between treatments in the same column followed by the same letter are not significantly different according to Tukey’s multiple range test (TMRT) at *p* ≤ 0.05. Specific effects are indicated with uppercase letters and the interactions are indicated with lowercase letters unless otherwise mentioned.

**Table 4:** Effects of soil addition of EM, compost tea, fulvic acid, yeast extract, and foliar spray with seaweed extract and their combination on fruit yield of sweet pepper plants during the 2019/2020 and 2020/2021 seasons.

Treatments		Number of Fruit/Plant		Total Fruit Yield (kg/m^2^)	
Potassium Soil Addition (S)	Foliar Spraying (F)	1st Season	2nd Season	1st Season	2nd Season
Control		14.9 ^C^	16.6 ^C^	11.31 ^C^	12.17 ^C^
EM		15.2 ^B^	16.8 ^C^	11.58 ^BC^	12.35 ^BC^
Compost tea		15.3 ^B^	17.6 ^AB^	12.41 ^A^	13.76 ^A^
Fulvic acid		15.7 ^A^	17.9 ^A^	12.34 ^A^	13.71 ^A^
Yeast extract		15.3 ^B^	17.3 ^B^	11.96 ^AB^	12.78 ^B^
	Control	14.8 ^C^	16.8 ^B^	10.85 ^D^	11.85 ^D^
	Seaweed at 1 g/L	15.2 ^B^	17.0 ^B^	11.66 ^C^	12.71 ^C^
	Seaweed at 2 g/L	15.4 ^B^	17.5 ^A^	12.21 ^B^	13.31 ^B^
	Seaweed at 3 g/L	15.8 ^A^	17.9 ^A^	12.97 ^A^	13.98 ^A^
Control	Control	14.3 ^h^	16.2 ^e^	10.29 ^h^	11.02 ^h^
	Seaweed at 1 g/L	14.8 ^f–h^	16.4 ^de^	11.19 ^d–h^	12.51 ^d–h^
	Seaweed at 2 g/L	15.1 ^d–g^	16.8 ^c–e^	11.86 ^c–g^	12.09 ^e–h^
	Seaweed at 3 g/L	15.4 ^b–f^	17.3 ^a–e^	11.90 ^c–g^	13.08 ^c–f^
EM	Control	14.6 ^gh^	16.4 ^de^	10.56 ^gh^	11.37 ^gh^
	Seaweed at 1 g/L	14.9 ^e–h^	16.3 ^de^	11.51 ^d–h^	11.68 ^fh^
	Seaweed at 2 g/L	15.7 ^a–d^	17.1 ^b–e^	11.97 ^c–g^	12.93 ^c–g^
	Seaweed at 3 g/L	15.6 ^a–e^	17.4 ^a–e^	12.31 ^b–f^	13.44 ^b–e^
Compost tea	Control	14.8 ^f–g^	16.9 ^b–e^	11.14 ^e–h^	12.22 ^d–h^
	Seaweed at 1 g/L	15.4 ^b–f^	17.6 ^a–d^	12.0 ^c–f^	13.36 ^c–e^
	Seaweed at 2 g/L	15.2 ^c–g^	18.0 ^a–c^	12.59 ^a–d^	14.38 ^ac^
	Seaweed at 3 g/L	16.1 ^ab^	18.2 ^ab^	13.92 ^a^	15.08 ^a^
Fulvic acid	Control	15.2 ^c–g^	17.6 ^a–d^	11.29 ^d–h^	12.55 ^d–h^
	Seaweed at 1 g/L	15.6 ^a–e^	17.9 ^a–c^	11.90 ^c–g^	13.65 ^a–e^
	Seaweed at 2 g/L	15.9 ^a–c^	18.1 ^a–c^	12.51 ^a–e^	13.74 ^a–d^
	Seaweed at 3 g/L	16.3 ^a^	18.6 ^a^	13.67 ^ab^	14.92 ^ab^
Yeast extract	Control	14.9 ^e–h^	17.1 ^b–e^	10.97 ^f–h^	12.08 ^e–h^
	Seaweed at 1 g/L	15.3 ^c–g^	17.0 ^b–e^	11.72 ^c–g^	12.23 ^d–h^
	Seaweed at 2 g/L	15.2 ^c–g^	17.6 ^a–d^	12.14 ^c–f^	13.42 ^b–e^
	Seaweed at 3 g/L	15.8 ^a–d^	17.8 ^a–c^	13.04 ^a–c^	13.40 ^b–e^

Means between treatments in the same column followed by the same letter were not significantly different according to Tukey’s multiple range test (TMRT) at *p* ≤ 0.05. Specific effects are indicated with uppercase letters and the interactions are indicated with lowercase letters unless otherwise mentioned.

**Table 5:** Effects of soil addition of EM, compost tea, fulvic acid, yeast extract, and foliar spray with seaweed extract and their combination on the physical quality of the fruit from sweet pepper plants during the 2019/2020 and 2020/2021 seasons.

Treatments		Average Fruit Fresh Weight (g)		Average Fruit Dry Weight (g)		Average Fruit Length (cm)		Average Fruit Diameter (cm)	
Potassium Soil Addition (S)	Foliar Spraying (F)	1st Season	2nd Season	1st Season	2nd Season	1st Season	2nd Season	1st Season	2nd Season
Control		229 ^D^	218 ^C^	16.8 ^A^	13.7 ^B^	8.18 ^C^	8.10 ^C^	7.66 ^B^	7.66 ^D^
EM		230 ^CD^	222 ^BC^	16.9 ^A^	14.0 ^B^	8.84 ^B^	8.33 ^BC^	8.41 ^A^	7.83 ^CD^
Compost tea		244 ^A^	235 ^A^	18.0 ^A^	14.8 ^A^	9.37 ^A^	9.26 ^A^	8.93 ^A^	8.87 ^A^
Fulvic acid		239 ^AB^	230 ^AB^	17.6 ^A^	14.8 ^A^	9.22 ^AB^	9.09 ^A^	8.78 ^A^	8.68 ^AB^
Yeast extract		236 ^BC^	222 ^BC^	17.4 ^A^	14.0 ^B^	8.90 ^D^	8.68 ^B^	8.49 ^B^	8.29 ^BC^
	Control	223 ^D^	213 ^C^	15.6 ^C^	12.8 ^D^	8.60 ^B^	8.52^A^	8.12 ^B^	8.07 ^A^
	Seaweed at 1 g/L	232 ^C^	223 ^B^	17.0 ^B^	14.3 ^C^	8.85 ^AB^	8.62 ^A^	8.38 ^AB^	8.19 ^A^
	Seaweed at 2 g/L	242 ^B^	230 ^AB^	18.1 ^A^	14.7 ^B^	9.04 ^A^	8.79 ^A^	8.58 ^A^	8.35 ^A^
	Seaweed at 3 g/L	248 ^A^	237 ^A^	18.8 ^A^	15.4 ^A^	9.15 ^A^	8.83 ^A^	8.74 ^A^	8.45 ^A^
Control	Control	218 ^h^	206 ^e^	15.2 ^f^	12.4 ^i^	7.93 ^e^	7.86 ^d^	7.12 ^c^	7.39 ^e^
	Seaweed at 1 g/L	229 ^f–h^	219 ^c–e^	16.7 ^b–f^	13.8 ^d–g^	8.07 ^de^	7.93 ^d^	7.61 ^bc^	7.54 ^de^
	Seaweed at 2 g/L	238 ^b–g^	218 ^c–e^	17.8 ^a–f^	14.0 ^d–f^	8.26 ^c–e^	8.31 ^b–d^	7.82 ^a–c^	7.82 ^b–e^
	Seaweed at 3 g/L	234 ^d–h^	229 ^a–d^	17.8 ^a–f^	14.9 ^b–d^	8.46 ^a–e^	8.28 ^cd^	8.09 ^a–c^	7.89 ^a–e^
EM	Control	219 ^h^	210 ^de^	15.3 ^f^	12.6 ^hi^	8.36 ^b–e^	8.21 ^cd^	8.02 ^a–c^	7.65 ^c–e^
	Seaweed at 1 g/L	23 4^d–h^	217 ^c–e^	17.1 ^a–f^	13.7 ^e–h^	8.80 ^a–e^	8.36 ^a–d^	8.32 ^a–c^	7.81 ^b–e^
	Seaweed at 2 g/L	231 ^e–h^	229 ^a–d^	17.3 ^a–f^	14.7 ^b–e^	9.12 ^a–e^	8.32 ^b–d^	8.70 ^ab^	7.86 ^a–e^
	Seaweed at 3 g/L	239 ^b–g^	234 ^a–c^	18.2 ^a–f^	15.2 ^a–c^	9.08 ^a–e^	8.40 ^a–d^	8.61 ^ab^	8.02 ^a–e^
Compost tea	Control	228 ^f–h^	219 ^c–e^	16.0 ^c–f^	13.1 ^f–i^	9.14 ^a–d^	9.06 ^a–c^	8.71 ^ab^	8.62 ^e^
	Seaweed at 1 g/L	236 ^c–g^	230 ^a–d^	17.3 ^a–f^	14.5 ^c–e^	9.23 ^a–d^	9.18 ^a–c^	8.83 ^ab^	8.76 ^a–d^
	Seaweed at 2 g/L	251 ^a–c^	242 ^ab^	18.8 ^a–d^	15.5 ^a–c^	9.52 ^ab^	9.37 ^ab^	8.96 ^ab^	9.0 ^ab^
	Seaweed at 3 g/L	262 ^a^	251 ^a^	19.9 ^a^	16.3 ^a^	9.61 ^a^	9.42 ^a^	9.24 ^a–c^	9.07 ^a^
Fulvic acid	Control	225 ^gh^	216 ^c–e^	15.8 ^d–f^	13.0 ^f–i^	8.93 ^a–e^	8.82 ^a–d^	8.51 ^ab^	8.41 ^a–e^
	Seaweed at 1 g/L	231 ^e–h^	231 ^a–d^	16.9 ^a–f^	15.8 ^ab^	9.17 ^a–d^	9.02 ^a–c^	8.64 ^ab^	8.63 ^a–e^
	Seaweed at 2 g/L	246 ^a–e^	230 ^a–d^	18.5 ^a–e^	14.7 ^b–e^	9.36 ^a–c^	9.20 ^a–c^	8.92 ^ab^	8.79 ^a–c^
	Seaweed at 3 g/L	254 ^ab^	243 ^ab^	19.3 ^ab^	15.8 ^ab^	9.42 ^a–c^	9.29 ^a–c^	9.06 ^a^	8.86 ^a–c^
Yeast extract	Control	223 ^g–h^	214 ^c–e^	15.6 ^ef^	12.8 ^g–i^	8.60 ^a–e^	8.64 ^a–d^	8.24 ^a–c^	8.26 ^a–e^
	Seaweed at 1 g/L	232 ^e–h^	218 ^c–e^	17.0 ^a–f^	13.7 ^e–h^	8.96 ^a–e^	8.61 ^a–d^	8.52 ^ab^	8.21 ^a–e^
	Seaweed at 2 g/L	242 ^b–f^	231 ^a–d^	18.2 ^a–f^	14.8 ^b–e^	8.92 ^a–e^	8.71 ^a–d^	8.51 ^ab^	8.28 ^a–e^
	Seaweed at 3 g/L	250 ^a–d^	228 ^b–e^	19.0 ^a–c^	14.8 ^b–e^	9.14 ^a–d^	8.73 ^a–d^	8.72 ^ab^	8.39 ^a–e^

Means between treatments in the same column followed by the same letter were not significantly different according to Tukey’s multiple range test (TMRT) at *p* ≤ 0.05. Specific effects are indicated with uppercase letters and the interactions are indicated with lowercase letters unless otherwise mentioned.

**Table 6:** Effects of soil addition of EM, compost tea, fulvic acid, yeast extract, and foliar spray with seaweed extract and their combination on the chemical quality of the fruit of sweet pepper plants during the 2019/2020 and 2020/2021 seasons.

Treatments		TSS (%)		VC (mg/100g f.w)		Total Sugars (%)		Carotenoids (mg/100g f.w)	
Soil Addition (S)	Foliar Spraying (F)	1st Season	2nd Season	1st Season	2nd Season	1st Season	2nd Season	1st Season	2nd Season
Control		5.28 ^B^	5.28 ^A^	106.9 ^C^	115.2 ^C^	3.34 ^B^	3.36 ^B^	0.86 ^B^	0.84 ^E^
EM		5.34 ^B^	5.34 ^A^	109.4 ^B^	120.0 ^B^	3.37 ^B^	3.37 ^B^	0.87 ^B^	0.85 ^D^
Compost tea		5.50 ^A^	5.39 ^A^	116.0 ^A^	123.2 ^B^	3.54 ^A^	3.47 ^AB^	0.91 ^A^	0.90^B^
Fulvic acid		5.55 ^A^	5.46 ^A^	119.9 ^A^	124.9 ^A^	3.60 ^A^	3.53 ^A^	0.94 ^A^	0.92 ^A^
Yeast extract		5.35 ^B^	5.35 ^A^	112.6 ^B^	118.2 ^B^	3.48 ^AB^	3.42 ^AB^	0.87 ^B^	0.88 ^C^
	Control	5.26 ^C^	5.26 ^A^	106.8 ^C^	113.0 ^C^	3.37 ^B^	3.35 ^B^	0.85 ^C^	0.85 ^D^
	Seaweed at 1 g/L	5.36 ^B^	5.34 ^A^	111.4 ^B^	117.1 ^B^	3.43 ^AB^	3.40 ^B^	0.88 ^BC^	0.87 ^C^
	Seaweed at 2 g/L	5.46 ^A^	5.39 ^A^	115.3 ^B^	124.2 ^B^	3.51 ^A^	3.44 ^AB^	0.90 ^AB^	0.89 ^B^
	Seaweed at 3 g/L	5.51 ^A^	5.46 ^A^	118.2 ^A^	126.8 ^A^	3.55 ^A^	3.50 ^A^	0.91 ^A^	0.92 ^A^
Control	Control	5.18 ^d^	5.21 ^a^	102.3 ^d^	106.3 ^d^	3.28 ^c^	3.31 ^a^	0.83 ^e^	0.81 ^n^
	Seaweed at 1 g/L	5.29 ^cd^	5.28 ^a^	108.3 ^c^	114.2 ^c^	3.32 ^bc^	3.38 ^a^	0.85 ^de^	0.83 ^l^
	Seaweed at 2 g/L	5.27 ^d^	5.29 ^a^	106.4 ^c^	121.2 ^b^	3.38 ^a–c^	3.34 ^a^	0.87 ^b–e^	0.83 ^l^
	Seaweed at 3 g/L	5.36 ^cd^	5.34 ^a^	110.3 ^b^	118.9 ^c^	3.37 ^a–c^	3.39 ^a^	0.88 ^a–e^	0.86 ^i^
EM	Control	5.21 ^d^	5.24 ^a^	104.7 ^d^	109.7 ^c^	3.31 ^bc^	3.36 ^a^	0.83 ^e^	0.82 ^m^
	Seaweed at 1 g/L	5.36 ^cd^	5.36 ^a^	104.2 ^e^	116.6 ^b^	3.39 ^a–c^	3.30 ^a^	0.89 ^a–e^	0.84 ^k^
	Seaweed at 2 g/L	5.41 ^b–d^	5.34 ^a^	112.6 ^a^	126.3 ^a^	3.36 ^a–c^	3.39 ^a^	0.88 ^a–e^	0.85 ^j^
	Seaweed at 3 g/L	5.35 ^cd^	5.41 ^a^	115.9 a	127.1 ^a^	3.42 ^a–c^	3.42 ^a^	0.87 ^a–e^	0.89 ^f^
Compost tea	Control	5.31 ^cd^	5.28 ^b^	108.6 ^b^	117.2 ^c^	3.41 ^a–c^	3.37 ^a^	0.86 ^c–e^	0.86 ^i^
	Seaweed at 1 g/L	5.38 ^cd^	5.34 ^a^	111.3 ^a^	121.3 ^a^	3.47 ^a–c^	3.42 ^a^	0.89 ^a–e^	0.89 ^f^
	Seaweed at 2 g/L	5.57 ^a–c^	5.42 ^a^	121.2 ^a^	125.4 ^a^	3.58 ^a–c^	3.51 ^a^	0.94 ^a–d^	0.92 ^d^
	Seaweed at 3 g/L	5.71 ^a^	5.49 ^a^	122.9 ^a^	128.9 ^a^	3.69 ^ab^	3.58 ^a^	0.95 ^a–c^	0.93 ^c^
Fulvic acid	Control	5.36 ^cd^	5.32 ^b^	112.2 ^c^	119.3 ^d^	3.48 ^a–c^	3.42 ^a^	0.89 ^a–e^	0.87 ^h^
	Seaweed at 1 g/L	5.42^b–d^	5.39 ^a^	118.3 ^b^	120.8 ^b^	3.53 ^a–c^	3.49 ^a^	0.92 ^a–e^	0.91 ^e^
	Seaweed at 2 g/L	5.69 ^ab^	5.52 ^a^	123.6 ^a^	128.3 ^a^	3.68 ^ab^	3.57 ^a^	0.96 ^ab^	0.94 ^b^
	Seaweed at 3 g/L	5.73 ^a^	5.61 ^a^	125.4 ^a^	131.2 ^a^	3.71 ^a^	3.62 ^a^	0.97 ^a^	0.95 ^a^
Yeast extract	Control	5.25 ^d^	5.2 6^a^	106.3 ^d^	112.6 ^c^	3.36 ^a-c^	3.39 ^a^	0.84 ^e^	0.87 ^h^
	Seaweed at 1 g/L	5.36 ^cd^	5.32 ^a^	114.8 ^b^	112.2 ^c^	3.42 ^a–c^	3.42 ^a^	0.84 ^e^	0.86 ^i^
	Seaweed at 2 g/L	5.34 ^cd^	5.38 ^a^	112.9 ^c^	119.8 ^b^	3.57 ^a–c^	3.39 ^a^	0.89 ^a–e^	0.89 ^f^
	Seaweed at 3 g/L	5.42 ^b–d^	5.43 ^a^	116.3 ^a^	128.1 ^a^	3.54 ^a–c^	3.48 ^a^	0.88 ^a–e^	0.88 ^g^

Means between treatments in the same column followed by the same letter were not significantly different according to Tukey’s multiple range test (TMRT) at *p* ≤ 0.05. Specific effects are indicated with uppercase letters and the interactions are indicated with lowercase letters unless otherwise mentioned.

**Table 7:** Physical and chemical analysis results for the experimental soil.

Physical Analysis		Chemical Analysis			
		Cations (meq/L)		Anions (meq/L)	
Coarse Sand	56.6%	Ca^++^	2.24	CO_3_^−^	Zero
Fine sand	29.4%	Mg^++^	1.86	HCO_3_**^−^**	3.00
Silt	5.0%	Na^+^	1.90	Cl**^−^**	1.70
Clay	9.0%	K^+^	0.50	SO_4_**^−^**	1.80
Texture class: sandy					
Soil pH	7.8	Available N: 2.28 mg/kg			
EC (dS/m)	0.65	Available P: 1.25 mg/kg			
Organic matter	0.71%	Available K: 11.9 mg/kg			

## Data Availability

Available upon request from the corresponding author.
